# Disentangling the Association of Hydroxychloroquine Treatment with Mortality in Covid-19 Hospitalized Patients through Hierarchical Clustering

**DOI:** 10.1155/2021/5556207

**Published:** 2021-06-25

**Authors:** Augusto Di Castelnuovo, Alessandro Gialluisi, Andrea Antinori, Nausicaa Berselli, Lorenzo Blandi, Marialaura Bonaccio, Raffaele Bruno, Roberto Cauda, Simona Costanzo, Giovanni Guaraldi, Lorenzo Menicanti, Marco Mennuni, Ilaria My, Giustino Parruti, Giuseppe Patti, Stefano Perlini, Francesca Santilli, Carlo Signorelli, Giulio Stefanini, Alessandra Vergori, Walter Ageno, Antonella Agodi, Piergiuseppe Agostoni, Luca Aiello, Samir Al Moghazi, Rosa Arboretti, Filippo Aucella, Greta Barbieri, Martina Barchitta, Paolo Bonfanti, Francesco Cacciatore, Lucia Caiano, Francesco Cannata, Laura Carrozzi, Antonio Cascio, Giacomo Castiglione, Arturo Ciccullo, Antonella Cingolani, Francesco Cipollone, Claudia Colomba, Crizia Colombo, Annalisa Crisetti, Francesca Crosta, Gian Battista Danzi, Damiano D'Ardes, Katleen de Gaetano Donati, Francesco Di Gennaro, Giuseppe Di Tano, Gianpiero D'Offizi, Francesco Maria Fusco, Carlo Gaudiosi, Ivan Gentile, Francesco Gianfagna1, Gabriele Giuliano, Emauele Graziani, Gabriella Guarnieri, Valerio Langella, Giovanni Larizza, Armando Leone, Gloria Maccagni, Federica Magni, Stefano Maitan, Sandro Mancarella, Rosa Manuele, Massimo Mapelli, Riccardo Maragna, Rossella Marcucci, Giulio Maresca, Silvia Marongiu, Claudia Marotta, Lorenzo Marra, Franco Mastroianni, Alessandro Mengozzi, Marianna Meschiari, Jovana Milic, Filippo Minutolo, Roberta Mussinelli, Cristina Mussini, Maria Musso, Anna Odone, Marco Olivieri, Antonella Palimodde, Emanuela Pasi, Raffaele Pesavento, Francesco Petri, Carlo A Pivato, Venerino Poletti, Claudia Ravaglia, Giulia Righetti, Andrea Rognoni, Marco Rossato, Ilaria Rossi, Marianna Rossi, Anna Sabena, Francesco Salinaro, Vincenzo Sangiovanni, Carlo Sanrocco, Nicola Schiano Moriello, Laura Scorzolini, Raffaella Sgariglia, Paola Giustina Simeone, Michele Spinicci, Enrica Tamburrini, Carlo Torti, Enrico Maria Trecarichi, Roberto Vettor, Andrea Vianello, Marco Vinceti, Agostino Virdis, Raffaele De Caterina, Licia Iacoviello

**Affiliations:** ^1^Mediterranea Cardiocentro, Napoli, Italy; ^2^Department of Epidemiology and Prevention, IRCCS Neuromed, Pozzilli, Italy; ^3^UOC Immunodeficienze Virali, National Institute for Infectious Diseases “L. Spallanzani” IRCCS, Rome, Italy; ^4^Section of Public Health, Department of Biomedical, Metabolic and Neural Sciences, University of Modena and Reggio Emilia, Modena, Italy; ^5^Università di Pavia, Pavia, Italy; ^6^Division of Infectious Diseases I, Fondazione IRCCS Policlinico San Matteo, Pavia, Italy; ^7^Department of Clinical, Surgical Diagnostic and Paediatric Sciences, University of Pavia, Pavia, Italy; ^8^Fondazione Policlinico Universitario A. Gemelli IRCCS, Roma, Italy; ^9^Università Cattolica Del Sacro Cuore- Dipartimento di Sicurezza e Bioetica Sede di Roma, Roma, Italy; ^10^Infectious Disease Unit, Department of Surgical, Medical Dental and Morphological Sciences, University of Modena and Reggio Emilia, Modena, Italy; ^11^IRCCS Policlinico San Donato, San Donato Milanese, Milan, Italy; ^12^University of Eastern Piedmont, Maggiore Della Carità Hospital, Novara, Italy; ^13^Humanitas Clinical and Research Hospital IRCCS, Rozzano, Milano, Italy; ^14^Department of Infectious Disease, Azienda Sanitaria Locale (AUSL) di Pescara, Pescara, Italy; ^15^Emergency Department, IRCCS Policlinico San Matteo Foundation, Pavia, Italy; ^16^Department of Internal Medicine, University of Pavia, Pavia, Italy; ^17^Department of Medicine and Aging, Clinica Medica, “SS. Annunziata” Hospital and University of Chieti, Chieti, Italy; ^18^School of Medicine, Vita-Salute San Raffaele University, Milano, Italy; ^19^HIV/AIDS Department, National Institute for Infectious Diseases Lazzaro SpallanzaniIRCCS, Roma, Italy; ^20^Department of Medicine and Surgery, University of Insubria, Varese, Italy; ^21^Department of Medical and Surgical Sciences and Advanced Technologies G.F. Ingrassia, University of Catania, AOU Policlinico G.Rodolico - San Marco, Catania, Italy; ^22^Centro Cardiologico Monzino IRCCS, Milano, Italy; ^23^Department of Clinical Sciences and Community Health, Cardiovascular Section, University of Milano, Milan, Italy; ^24^UOC. Anestesia e Rianimazione, Dipartimento di Chirurgia Generale Ospedale Morgagni-Pierantoni, Forlì, Italy; ^25^UOC Infezioni Sistemiche Dell'Immunodepresso, National Institute for Infectious Diseases L. Spallanzani IRCCS, Rome, Italy; ^26^Department of Civil Environmental and Architectural Engineering, University of Padova, Padova, Italy; ^27^Fondazione I.R.C.C.S Casa Sollievo Della Sofferenza, San Giovanni Rotondo, Foggia, Italy; ^28^Department of Surgical Medical and Molecular Medicine and Critical Care, Azienda Ospedaliera Universitaria Pisana and University of Pisa, Pisa, Italy; ^29^Department of Medical and Surgical Sciences and Advanced Technologies G.F. Ingrassia, University of Catania, Catania, Italy; ^30^UOC Malattie Infettive, Ospedale San Gerardo, ASST Monza, Monza, Italy; ^31^School of Medicine and Surgery, University of Milano-Bicocca, Milano, Italy; ^32^Department of Translational Medical Sciences, University of Naples, Federico II, Naples, Italy; ^33^Cardiovascular and Thoracic Department, Azienda Ospedaliero-Universitaria Pisana and University of Pisa, Pisa, Italy; ^34^Infectious and Tropical Diseases Unit- Department of Health Promotion, Mother and Child Care, Internal Medicine and Medical Specialties (PROMISE) - University of Palermo, Palermo, Italy; ^35^Servizio di Anestesia e Rianimazione II UO Rianimazione Ospedale San Marco, AOU Policlinico G. Rodolico, San Marco, Catania, Italy; ^36^Department of Cardiology, Ospedale di Cremona, Cremona, Italy; ^37^Medical Direction IRCCS Neuromed, Pozzilli, Italy; ^38^UOC Malattie Infettive-Epatologia, National Institute for Infectious Diseases L. Spallanzani IRCCS, Roma, Italy; ^39^UOC Infezioni Sistemiche e Dell'Immunodepresso, Azienda Ospedaliera Dei Colli Ospedale Cotugno, Napoli, Italy; ^40^ASL Napoli3 Sud COVID HOSPITAL, Boscotrecase, Napoli, Italy; ^41^Department of Clinical Medicine and Surgery, University of Naples Federico II, Napoli, Italy; ^42^Medicina Interna, Ospedale di Ravenna, AUSL Della Romagna, Ravenna, Italy; ^43^Respiratory Pathophysiology Division, Department of Cardiologic, Thoracic and Vascular Sciences, University of Padova, Padova, Italy; ^44^UOC Medicina COVID- PO S. Maria di Loreto Nuovo, ASL Na 1 Centro, Napoli, Italy; ^45^COVID-19 Unit. EE Ospedale Regionale F. Miulli, Acquaviva Delle Fonti, Bari, Italy; ^46^UOC di Pneumologia P.O. San Giuseppe Moscati, Taranto, Italy; ^47^ASST Milano Nord - Ospedale Edoardo Bassini, Cinisello Balsamo, Italy; ^48^U.O. C. Malattie Infettive e Tropicali, P.O. “San Marco” AOU Policlinico “G. Rodolico - San Marco”, Catania, Italy; ^49^Department of Experimental and Clinical Medicine, University of Florence and Azienda Ospedaliero-Universitaria Careggi, Firenze, Italy; ^50^Santissima Trinità di Cagliari, Cagliari, Italy; ^51^Department of Clinical and Experimental Medicine, Azienda Ospedaliera Universitaria Pisana University of Pisa, Pisa, Italy; ^52^Dipartimento di Farmacia, Università di Pisa, Pisa, Italy; ^53^UOC Malattie Infettive-Apparato Respiratorio, National Institute for Infectious Diseases L. Spallanzani IRCCS, Rome, Italy; ^54^Computer Service, University of Molise, Campobasso, Italy; ^55^Clinica Medica 3. Department of Medicine - DIMED, University Hospital of Padova, Padova, Italy; ^56^UOC Pneumologia. Dipartimento di Malattie Apparato Respiratorio e Torace, Ospedale Morgagni-Pierantoni Forlì, Forlì, Italy; ^57^Department of Respiratory Diseases & Allergy Aarhus University Hospital, Aarhus, Denmark; ^58^UOC Malattie Infettive Ad Alta Intensità di Cura, National Institute for Infectious Diseases L. Spallanzani IRCCS, Rome, Italy; ^59^Infectious and Tropical Diseases Unit, Deparment of Medical and Surgical Sciences, Magna Graecia University, Catanzaro, Italy; ^60^Department of Epidemiology, Boston University School of Public Health, Boston, USA

## Abstract

The efficacy of hydroxychloroquine (HCQ) in treating SARS-CoV-2 infection is harshly debated, with observational and experimental studies reporting contrasting results. To clarify the role of HCQ in Covid-19 patients, we carried out a retrospective observational study of 4,396 unselected patients hospitalized for Covid-19 in Italy (February–May 2020). Patients' characteristics were collected at entry, including age, sex, obesity, smoking status, blood parameters, history of diabetes, cancer, cardiovascular and chronic pulmonary diseases, and medications in use. These were used to identify subtypes of patients with similar characteristics through hierarchical clustering based on Gower distance. Using multivariable Cox regressions, these clusters were then tested for association with mortality and modification of effect by treatment with HCQ. We identified two clusters, one of 3,913 younger patients with lower circulating inflammation levels and better renal function, and one of 483 generally older and more comorbid subjects, more prevalently men and smokers. The latter group was at increased death risk adjusted by HCQ (HR[CI95%] = 3.80[3.08-4.67]), while HCQ showed an independent inverse association (0.51[0.43-0.61]), as well as a significant influence of cluster∗HCQ interaction (*p* < 0.001). This was driven by a differential association of HCQ with mortality between the high (0.89[0.65-1.22]) and the low risk cluster (0.46[0.39-0.54]). These effects survived adjustments for additional medications in use and were concordant with associations with disease severity and outcome. These findings suggest a particularly beneficial effect of HCQ within low risk Covid-19 patients and may contribute to clarifying the current controversy on HCQ efficacy in Covid-19 treatment.

## 1. Introduction

Hydroxychloroquine (HCQ) is an antimalarial drug suggested to be effective in inhibiting Severe Acute Respiratory Syndrome Coronavirus 2 (SARS-Cov-2) replication in vitro [1, 2]. Indeed, HCQ is characterized by antiviral, anti-inflammatory, and antithrombotic actions, contrasting the main disruptive effects of SARS-CoV-2 infection on the organism [3]. For this reason, it has been heavily used in treating patients affected by SARS-CoV-2 infection related disease (commonly known as Covid-19), especially in the first phases of the current pandemics, when Covid-19 was quite unknown [4].

Despite these elements and initial suggestive evidence of efficacy based on daily clinical practice, in the last months, the potential benefit of HCQ for Covid-19 patients has been harshly debated [3, 5]. In particular, evidence supporting protective effects from observational studies [6–13] was in contrast with that suggesting no effect at all by recent randomized clinical trials (RCTs) [14–18]. More recently, a meta-analysis combining both RCTs and observational studies over more than 44,000 patients supported a protective effect of HCQ, driven by the findings of observational studies [5]. A potential explanation for this discrepancy may be due to the usually high dosage administered in RCTs (800 mg/day), compared to lower dosages reported in observational studies supporting HCQ efficacy (≤400 mg/day), as hypothesized elsewhere [5]. As an alternative explanation, it is likely that the efficacy of HCQ treatment for Covid-19 may vary across patients and is influenced by subtypes of the disease, which in turn is largely dependent on patients' characteristics and their nonlinear combinations [19]. In this “personalized medicine” view, response to HCQ may not be the same across all patients of the same age, or with similar circulating inflammation levels. In order to identify these combinations, the use of big data like Electronic Health Records (EHRs) and of machine learning (ML) algorithms to interpret hidden HCQ response patterns is of fundamental importance. ML is an umbrella term covering different algorithms designed for the identification of hidden patterns, information mining, and variable classification/estimation through modelling complex (including nonlinear) functions, usually adopted in a big data setting. These algorithms can be generally classified into supervised and unsupervised approaches. The formers are designed to learn to predict specific outcomes after proper training of the algorithm in independent datasets, trying to model relationships and dependencies between the input variables (or features) and the target prediction output (or label). In unsupervised algorithms, the machine simply learns to identify hidden patterns across a high number of observations over many features, without the need for labels, and is more descriptive—rather than predictive—in nature. These algorithms have shown promising findings in public health, in the management of both chronic [20] and acute health conditions [21, 22], but also during the current pandemics. In particular, useful ML applications have been reported in the prediction of Covid-19 diagnosis and prognosis [23]. Notwithstanding this, to the best of our knowledge, only one study attempted so far to predict response to HCQ treatment within Covid-19 patients, through the application of a supervised ML technique (gradient boosting). Interestingly, the authors reported a reduction of in-hospital mortality within patients treated with HCQ, which was even more pronounced within those patients predicted to benefit most from the drug, in line with expectations [19].

Here, we attempted a personalized Covid-19 patient characterization to better disentangle the beneficial effects of HCQ previously reported within the COVID-19 RISK and Treatments (CORIST) study, a large retrospective cohort of patients hospitalized for SARS-CoV-2 infection in Italy [13]. This approach consisted of i) identifying the existence of subtypes of Covid-19 patients through an unsupervised ML algorithm—hierarchical clustering—comparing their characteristics and their clinical risks, ii) testing the resulting patients' clusters for association with mortality and modification of effect by treatment with HCQ, and iii) analyzing potential interactions between clusters and HCQ use. This approach represents a prominent example of how personalized medicine may support clinicians in Covid-19 treatment.

## 2. Methods

### 2.1. Analyzed Cohort

The COVID-19 RISK and Treatments (CORIST) study includes 4,396 patients hospitalized for SARS-Cov-2 infection in 35 hospitals across Italy, between February 2020 and May 2020. Molecular diagnosis of SARS-CoV-2 infection was based on polymerase chain reaction (PCR) of viral DNA extracted and amplified from nasopharyngeal swabs. Within each participating hospital, clinical data were abstracted at one-time point from electronic medical records or charts and collected using either a centrally designed electronic worksheet or a centralized web-based database. Collected data included patients' demographics, laboratory tests, medications in use, history of disease, and prescribed pharmacological therapy for Covid-19 treatment. For each participant, the study index date was defined as the date of hospital admission, while the study end point was death. Follow-up time was computed as the time between the index date and death, or alternatively between the index date and the date of discharge, applying right-censoring. Further details on the study are reported elsewhere [13, 24, 25].

### 2.2. Statistical Analyses

#### 2.2.1. Cluster Analysis

All analyses were carried out in *R* 4.0.2 (https://www.r-project.org/) [26]. We applied a hierarchical clustering analysis on Covid-19 patients using their main clinical, lifestyles, and sociodemographic characteristics, which were suggested as the most influential on mortality risk by previous studies in the field [25, 27, 28]. These included age (years), sex, glomerular filtration rate (eGFR, mL/min/1.73 m^2^) and high-sensitivity plasma C-reactive protein levels (mg/L) at in-hospital admission, obesity (body mass index (BMI) ≥ 30 kg/m^2^), hypertension (Yes/No), and smoking status (never/previous/current smoker), as well as history of myocardial infarction, heart failure, chronic pulmonary disease, cancer, and diabetes (Yes/No). Missing data were imputed through a k-Nearest Neighbor approach, implemented in the *knn()* function of the VIM package (version 6.1.0; https://cran.r-project.org/web/packages/VIM/index.html), with *k* = 10 [29]. CRP was transformed on the natural logarithm scale to reduce skewness, and all the continuous variables were normalized through the *normalize()* function in Keras v2.3.0 (https://cran.r-project.org/web/packages/keras/index.html).

Cluster analyses were then performed on the 4,396 patients, using the variables specified above, through the Cluster package v2.1.0 (https://cran.rproject.org/web/packages/cluster/index.html). First, we computed a dissimilarity matrix based on Gower pairwise distance (Figure S1), through the *daisy()* function. Gower distance is a parameter in the [0;1] range representing the average of partial dissimilarities across individuals (the higher the distance, the more the dissimilarities for a given pair of subjects) [30]. Second, we performed hierarchical clustering through the *hclust()* function applied to the Gower distance matrix computed above, which separated subjects based on their degree of pairwise dissimilarity, both from lowest to highest (agglomerative clustering) and from highest to lowest (divisive clustering). Third, we determined the appropriate number of clusters for patient classification, based on the Average Silhouette method (Figure S2). This computes the number of clusters, which maximizes the average silhouette width, a measure of the quality of clustering indicating how well each object lies within its cluster [31]. This method, applied through the *fviz_nbclust()* function of the Factoextra package (v1.0.7, https://cran.rproject.org/web/packages/factoextra/index.html), computed *k* = 2 as the optimal number of clusters. Finally, each patient was assigned to one of the two clusters determined above, through application of the *cutree()* function (Cluster package) to the results of the cluster analysis previously carried out.

#### 2.2.2. Comparison of Clusters

First, we compared the classifications made through agglomerative and hierarchical clustering, which revealed high consistency (Odds Ratio = 34.0 [26.7-43.7], Fisher Exact Test *p* value < 10^−15^). In light of this homogeneity of classification, and since divisive clustering has been reported to be more accurate and robust [32], all the subsequent analyses were performed on cluster classification identified through the latter approach.

The two clusters of patients identified were then compared for all anamnestic variables mentioned above, through Fisher's Exact Test (for binary variables), Chi-squared test (for nonbinary categorical variables), and through Student's *t*-test or Wilcoxon Rank Sum tests (for continuous variables meeting and not meeting parametric assumptions, respectively). Similarly, we compared Covid-19 disease severity, classified by recruiting centers in asymptomatic/mild, nonsevere pneumonia, severe pneumonia, and acute respiratory distress syndrome (ARDS). Moreover, we compared the use of six common drugs for Covid-19 treatment between the two clusters, including hydroxychloroquine, antihypertensive drugs, anti-interleukin-6 antibody, antivirals (Remdesivir, Lopinavir, Darunavir), and corticosteroids. These were reported as binary variables (Yes/No) and were, therefore, compared with clusters through Fisher Exact Tests.

#### 2.2.3. Survival Analyses

Once the clusters were characterized, we modelled incident mortality risk as a function of patients clusters and use of HCQ through Cox Proportional Hazards (PH) models, using the *cox.zph()* function of the survival package (v3.2.7, see URLs: https://cran.r-project.org/web/packages/survival/index.html) [33]. Only patients with complete information needed in each model were included in the analysis (case-complete approach; see below). A preliminary check of the basic Cox PH assumptions revealed no influential observations based on dfbeta residuals (Figure S3a), while Schoenfeld residuals tests revealed a statistical violation of the proportionality of hazards assumption, although these did not show any evident trend at a visual inspection (Figure S3b). For this reason, we carried out Cox PH models both with and without including an interaction term with time-to-event, a strategy commonly used to overcome this violation [34]. Incrementally adjusted models were analyzed: i) a crude model including only patients' clusters (Model 1; *N* = 4,319); ii) a model testing additive influence of clusters and HCQ use (Model 2: Model 1 + HCQ; *N* = 4,212); and iii) a model testing both additive and synergistic influence of clusters and HCQ use (Model 3: Model 2 + clusters∗HCQ; *N* = 4,212). Additional sensitivity analyses were carried out to rule out potential confounding effects of additional drugs in use for Covid-19 treatment (Model 4: Model 3 + other drugs). Risk estimates were computed as hazard ratios (HR) with 95% confidence intervals (95% CI) of dying, and HR with *p* values below *α* = 0.05 were considered significant. To quantify the potential for unmeasured confounding effects, the E-value was calculated for all the HRs observed in Model 3, as described in [35] (https://www.evalue-calculator.com/). This represents the minimum association required for a potential unmeasured confounder with both the exposure and the outcome to explain away the observed association. In other words, the higher the E-value, the harder it is to attribute an association to an unmeasured covariate [36].

#### 2.2.4. Associations with Additional Endpoints

To better evaluate the associations of Covid-19 patients clusters and HCQ use with negative outcomes other than death, we built a composite endpoint based on the occurrence of at least one of the following outcomes: in-hospital death, access to intensive care unit during hospitalization, or severe disease manifestation (either severe pneumonia or ARDS). In this case, the resulting binary variable was assigned a value of 1. Conversely, the variable got “0” value if one of the following alternative conditions is applied: (i) none of the above mentioned outcomes was verified; (ii) a patient survived without recurring to intensive cares or (iii) without showing severe manifestations of the disease. Six patients with missing values on survival were removed. Then, we modelled the risk of manifesting a bad outcome through a logistic regression (*glm()* function in R), modelling both additive and interactive models of Covid-19 patients cluster and HCQ use, as above. This analysis was motivated by the fact that the curse of disease often differs across patients, e.g., with some subjects with less severe forms suddenly worsening their conditions until death and others having severe manifestations but still surviving, possibly thanks to intensive cares. Therefore, a composite outcome variable represented a robust way to measure potential risk/protective effects of patients' clusters and HCQ use.

## 3. Results

While both agglomerative and divisive clustering approaches were developed, all statistical analyses presented below are based on clusters identified through the latter approach, since this showed a high homogeneity with the results of agglomerative clustering (see Methods section), and divisive clustering has been reported to be more accurate and robust [32, 34].

### 3.1. Characteristics of the Clusters

**Figure 1:**
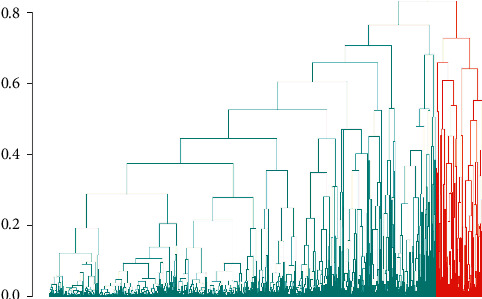
Hierarchical divisive clustering of Covid-19 hospitalized patients. Two main clusters of patients were identified, with *N* = 3,913 (green) and 483 (red), respectively. Each line on the *x* axis represents a patient, while on the *y* axis the Gower distance between patients is reported. The higher the distance, the later the two patients join into a subcluster, and the more dissimilar they are.

**Figure 2:**
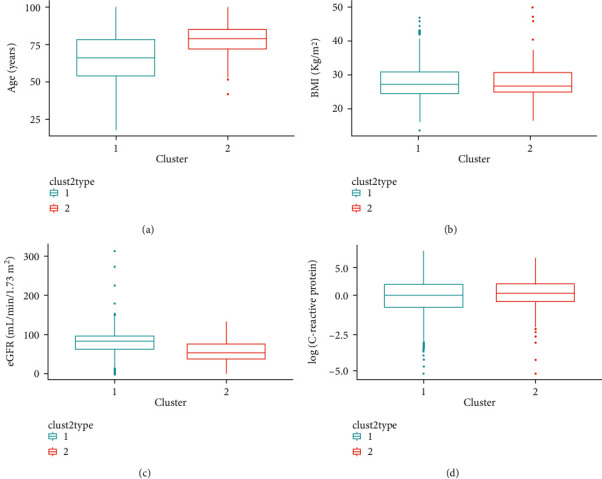
Characteristics of sample according to the two clusters identified.Comparison of the continuous variables used for hierarchical clustering—including (a) age (years), (b) BMI (Kg/m^2^), (c) eGFR (mL/min/1.73 m^2^), and (d) C-reactive protein plasma levels (mg/L, log-scale) between the two clusters of Covid-19 patients identified, namely, the low (green) and the high risk (red) cluster. Here, these variables are represented through boxplots, with boxes showing the interquartile ranges (IQR = *Q*1-Q3), continuous lines showing the whole distribution range from Q1 – 1.5∗IQR through *Q*3 + 1.5∗IQR, and dots showing more extreme values in the dataset.

We identified two clusters of Covid-19 patients (with *N* = 3,913 and 483, Figure 1). A comparison of the continuous variables used for their determination is reported in Figures 2(a)–2(d). The larger cluster was younger (mean (SD) age: 65.2 (15.6) vs 77.9 (9.2) years; *t*-test = -25.9, *p* < 10^−15^), with better kidney function (eGFR: 77.9 (26.9) vs 52.6 (25.6) mL/min/1.73 m^2^; *t*-test = 20.3, *p* < 10^−15^) and lower circulating inflammation levels (CRP: 34.6 (62.4) vs 36.5 (58.6) mg/L; Wilcoxon-test = 847,660, *p*=2.5 × 10^−14^). Conversely, BMI did not show strong differences between the two clusters (28.0 (4.2) vs 27.5 (4.2) Kg/m^2^; *t*-test = 2.3, *p*=0.02). Moreover, patients belonging to the larger cluster were less frequently men (60% vs 75%) and smokers (11.5% vs 19.5%) and showed a lower prevalence of chronic health conditions like myocardial infarction, heart failure, diabetes, hypertension, cancer, and lung disease (all *p* < 0.0001), while no significant difference was observed in the prevalence of obesity (Table 1).

Clusters were also associated with severe Covid-19 disease manifestations, with 65.8% of patients in the smaller cluster presenting with either severe pneumonia or ARDS, compared to 45.9% in the larger cluster (Chi-squared = 76.4, *p* < 10^−15^; Table S1). For the characteristics mentioned above, the large and small clusters will be hereafter named as “low risk” and “high risk” cluster. When we compared the use of specific drugs, in the high risk cluster, we observed a less frequent use of HCQ (*p* < 0.001) and of Lopinavir/Darunavir (*p* < 0.05) and a more frequent use of corticosteroids and antihypertensive medications (*p* < 0.001), compared to the low risk cluster (Table S2).

### 3.2. Combined Influence of Clusters and HCQ Use on Mortality Risk

In Cox PH regressions modelling mortality risk as a function of clusters (Model 1), we analyzed 4,319 patients with a case-complete approach, with a total of 799 deaths and a total of 73,924 person-days follow-up (median 13 days). In this model, patients belonging to the high risk cluster showed a significant increase of incident mortality risk, compared to those of the low risk cluster (HR [CI] = 3.81 [3.12-4.65]; Table 2). This association remained stable in a Cox regression modelling additive effects of clusters and HCQ use (Model 2: *N* = 4,212, 743 events, a total of 72,239 person-days follow-up, median 14 days). Indeed, the high risk cluster was associated with a significant increase of mortality (3.80 [3.08-4.67]), while HCQ use was associated with a significant independent reduction (0.51 [0.43-0.61]). When we modelled additive and interactive associations of clusters and HCQ in a single model (Model 3), we observed a substantially stable protective association of HCQ (0.46 [0.38-0.55]), a reduced but still significant direct association of the “high risk” cluster (2.45 [1.69-3.54]), and a significant association of the cluster∗HCQ interaction term with incident mortality (*p*=2.1 × 10^−4^). This was driven by a differential association of HCQ use within the different clusters, since this was associated with a notable reduction of mortality risk in the low risk cluster (HR [CI] = 0.46 [0.39-0.54], *p* < 10^−15^) and with a milder nonsignificant reduction in the high risk cluster (0.89 [0.65-1.22], *p*=0.47). The abovementioned associations were quite robust against potential unmeasured cofounding effects, with E-values of 3.1, 2.8, and 2.5 for the associations of mortality with the risk cluster, HCQ use, and cluster∗HCQ interaction in Model 3. Moreover, these associations remained substantially stable in Cox PH models including additional drugs in use (Table 2, Model 4), as well as in those including an interaction term with time (Table S3).

### 3.3. Associations with a Combined Covid-19 Outcome

When we modelled the risk of bad clinical outcomes of the disease—i.e., severe Covid-19 manifestations, access to intensive care unit or death—as a function of clusters and HCQ use, we observed results in line with survival analyses, with increased risk for cluster 2 and decreased risk for HCQ users, both in the additive and in the interactive model (Table 3). While the cluster∗HCQ interaction showed only a trend of significance (*p*=0.08), HCQ still presented a significant protective association in the low risk cluster (OR [CI] = 0.67 [0.56-0.79], *p*=4.0 × 10^−6^) and a substantially null association in the high risk cluster (OR [CI] = 0.98 [0.66-1.46], *p*=0.92).

## 4. Discussion

In the present work, we report differential influence of HCQ treatment on Covid-19 mortality through a hierarchical clustering analysis applied to patients hospitalized for SARS-CoV-2 infection. This revealed the existence of two separate clusters of Covid-19 patients, based on their clinical and sociodemographic characteristics: one of younger patients with less comorbidities, lower circulating inflammation, and better renal function (“low risk” cluster), and one of older and more comorbid patients, more prevalently men and smokers (“high risk” cluster). The former cluster showed a higher prevalence of severe manifestations of Covid-19, ranging from severe pneumonia to ARDS. Moreover, survival analyses showed an almost four-fold increase of incident in-hospital mortality for the high risk compared to the low risk cluster. Although a previous study attempted to identify subtypes of Covid-19 patients, associating them with disease severity [37], this represents the first attempt to use clustering in disentangling the effect of HCQ on different types of patients, by testing associations with incident in-hospital mortality risk. Specifically, we tested and observed both additive and interactive associations of HCQ and Covid-19 subtypes. Indeed, the high risk cluster was consistently associated with increased mortality across all models, while treatment with HCQ was generally associated with a halving of death risk, in line with previous evidence from both observational [6–13] and experimental studies [19]. While we already reported evidence suggesting a protective influence of HCQ against mortality in a largely overlapping sample [13], here, we have further deepened this relationship by testing and reporting a significant association between cluster-by-HCQ interaction and mortality, which was driven by a differential association within the two clusters. Indeed, the low risk cluster showed a significant “protective” influence of HCQ on in-hospital deaths, while the high risk cluster showed a concordant but nonsignificant association. This represents an element of novelty of the present study, since, in our previous work, we observed a “protective” association between HCQ and mortality within the totality of patients (about 75% of the current sample size), and when stratifying by age, sex, and other characteristics [13], but not within different subtypes of patients combining all these characteristics together in a nonlinear setting, as can be built through unsupervised ML algorithms. Moreover, here, our evidence is supported also by concordant associations with a composite and possibly more robust outcome of the disease, based on the occurrence of death, access to ICU, and severity of manifestations.

Recently, an approach based on the definition of subtypes of Covid-19 patients has been already proven to be successful in identifying which patients benefit most from HCQ treatment, in a multicenter trial involving six US hospitals and 290 patients hospitalized for Covid-19, the IDENTIFY study [19]. HCQ treatment was associated with higher survival in the treated harm, and especially within those patients that were predicted to benefit most based on a supervised ML algorithm applied to their characteristics, which included blood pressure, heart rate, temperature, respiratory rate, oxygen saturation, white blood cell and platelet count, lactate, blood urea nitrogen, creatinine, and bilirubin levels [19]. Interestingly, lactate and creatinine levels were the most important features in this algorithm [19], the latter representing an index of renal function, which was also a characteristic feature of the low risk cluster in the present study, where HCQ was more effective. Moreover, patients eligible for HCQ treatment as derived by the algorithm of [19] were shown to be younger and less comorbid than the whole population studied, in line with the evidence reported in the present work, suggesting that HCQ treatment may be more effective for younger patients with better general health conditions.

### 4.1. Strengths and Limitations

Although, to the best of our knowledge, this study represents the largest and broadest cluster analysis on Covid-19 patients and a novel approach in analyzing the influence of a pharmacological treatment on Covid-19 mortality and outcomes, it also presents few limitations.

First, the observational retrospective design does not allow us to completely control for confounders and randomization of treatments across individuals. The former aspect is quite unlikely, since a potential residual confounder should be strongly associated with in-hospital mortality to take away observed associations in the interactive model, as suggested by the computed E-values [35, 36]. As for drug therapy, we cannot rule out that assignment to specific treatments was driven also by clinical conditions of the participants, as usually found in common clinical practice. For the same reason, the protective association observed for HCQ may be hypothesized to be driven by other coadministered medications. However, here, HCQ and patients' cluster showed significant independent associations, which remained substantially stable across models and survived correction for other drugs in use for Covid-19 treatment. Lastly, our evidence is in contrast with RCTs published so far [14–18], which are commonly conceived as the gold standard for establishing drug efficacy and safety. While we generally agree with this view, we would like to underline that these studies did not randomize patients to treatment arms based on combinations of their features, but rather based on single characteristics such as age and sex. This may be the reason for this discrepancy, along with the hypothesis that the high dosage of HCQ administered in RCTs may be harmful for patients, compared to lower dosages reported in observational studies supporting HCQ efficacy [5]. Of interest, a recent critical review underlined the aspect of suboptimal randomization methods of RCTs, which often do not take into account the whole patient profile and disease severity and may lead to misleading conclusions [38].

## 5. Conclusions

Overall, the evidence supported here and elsewhere [19] suggests that HCQ treatment may be more effective in specific subtypes of Covid-19 patients and indicates machine learning as a useful approach to identify the most “promising” patients in terms of success rate of this treatment. In the future, further studies on independent datasets are warranted, possibly using supervised ML techniques as in other clinical settings (e.g., [39, 40]), to validate this hypothesis and test the feasibility of predicting responsiveness to HCQ before intervention. Ideally, a trial administering low dosages of HCQ (≤400 mg/day) and randomizing subjects based on their Covid-19 subtype profile rather than on single characteristics may be warranted to clarify the effects of HCQ on mortality risk in SARS-CoV-2 infection, especially within those patients with a “low risk” profile.

This may help solving current controversies on the use of HCQ as a medication for Covid-19 and maximize the efficacy of treatment strategies for this yet largely unknown disease, especially in low-income and developing countries with poorer national health systems.

## Figures and Tables

**Table 1:** Comparison of main categorical variables between the two clusters identified.

Category (%)	Cluster 1 – low risk *N* = 3,913	Cluster 2 – high risk *N* = 483	p for difference
Men	2,346 (60.0%)	362 (74.9%)	6 × 10^−11^
Smoke			<10^−15^
Current smokers	450 (11.5%)	94 (19.5%)	
Previous smokers	268 (6.8%)	94 (25.3%)	
Obesity (BMI ≥ 30 Kg/m^2^)	546 (13.9%)	64 (13.3%)	0.73
Myocardial infarction	127 (3.2%)	335 (69.4%)	<10^−15^
Heart failure	171 (4.4%)	315 (65.2%)	<10^−15^
Diabetes	621 (15.9%)	276 (57.1%)	<10^−15^
Hypertension	1,828 (46.7%)	453 (93.8%)	<10^−15^
Cancer	392 (10.0%)	89 (18.4%)	2 × 10^−07^
Lung disease	415 (10.6%)	207 (42.8%)	<10^−15^

P for difference resulting from comparison of the clusters—through Fisher's Exact Test (for binary variables) or Chi-squared test (for nonbinary categorical variables, i.e., smoke)—are reported, along with absolute and % frequency of each condition within each cluster.

**Table 2:** Results of Cox PH regressions modelling incident mortality risk.

Model	N (deaths)	Cluster 2 vs 1	HCQ Yes vs no	Cluster∗HCQ
Model 1: Death ∼ cluster	4,319 (799)	**3.81 [3.12-4.65] (<10** ^**−15**^ **)**		

Model 2: Death ∼ cluster + HCQ	4,212 (743)	**3.80 [3.08-4.67] (<10** ^**−15**^ **)**	**0.51 [0.43-0.61] (8.8 × 10** ^**−15**^ **)**	

Model 3: Death ∼ cluster + HCQ + Cluster∗HCQ	4,212 (743)	**2.45 [1.69-3.54] (4.9 × 10** ^**−4**^ **)**	**0.46 [0.38-0.55] (<10** ^**−15**^ **)**	**1.90 [1.21-2.96] (2.1 × 10** ^**−4**^ **)**

Model 4: Death ∼ cluster + HCQ + Cluster∗HCQ + other drugs	3,736 (664)	**1.65 [1.20-2.26] (2.2 × 10** ^**−3**^ **)**	**0.52 [0.43-0.63] (2.0 × 10** ^**−11**^ **)**	**1.98 [1.36-2.89] (4.0 × 10** ^**−4**^ **)**

Associations between incident mortality risk, Covid-19 clusters identified, and use of Hydroxychloroquine (HCQ) were tested in the incremental models and in a sensitivity analysis including all the drugs used for Covid-19 treatment. No other covariates were included in the analysis. Hazard Ratios with 95% confidence intervals (HR [CI]) and relevant *p*-values (in brackets) are reported. Significant HRs (*p* < 0.05) are highlighted in bold.

**Table 3:** Results of logistic regressions modelling Covid-19 composite bad outcome risk.

Model	N	Cluster 2 vs 1	HCQ	Cluster∗HCQ
Bad outcome ∼ cluster	4,373	**2.53 [2.09-3.08] (<10** ^**−15**^ **)**		

Bad outcome ∼ cluster + HCQ	4,265	**2.53 [2.08-3.09] (<10** ^**−15**^ **)**	**0.71 [0.61-0.83] (2.1 × 10** ^**−5**^ **)**	

Bad outcome ∼ cluster + HCQ + Cluster∗HCQ	4,265	**1.94 [1.35-2.79] (3.8 × 10** ^**−4**^ **)**	**0.67 [0.56-0.79] (4.0 × 10** ^**−6**^ **)**	1.46 [0.95-2.26] 0.08

Bad outcome ∼ cluster + HCQ + Cluster∗HCQ + other drugs	3,786	**1.80 [1.21-2.68] (3.8 × 10** ^**−3**^ **)**	**0.55 [0.45-0.66] (9.0 × 10** ^**−10**^ **)**	1.47 [0.92-2.36] (0.10)

The composite bad outcome was defined as one of the following: death, access to intensive care unit, or severe Covid-19 manifestation (either severe pneumonia or ARDS). Associations were tested in three incremental models and in a sensitivity analysis including all the drugs used for Covid-19 treatment, as for Cox PH regressions. Odds Ratios with 95% confidence intervals (OR [CI]) and relevant *p*-values (in brackets) are reported. Significant ORs (*p* < 0.05) are highlighted in bold.

## Data Availability

The data used to support the findings of this study may be released upon application to the CORIST collaboration board, who can be contacted through corresponding author.

## Supplementary Materials

Supplementary Materials Figure S1. Cross-patient dissimilarity matrix based on Gower distance. Figure S2. Optimal number of clusters (k) to apply in the hierarchical clustering analysis. Figure S3. Check for basic assumptions of Cox PH models. Table S1. Contingency table of Covid-19 cluster by disease severity. Table S2. Contingency tables of Covid-19 clusters by drugs used for treatment. Table S3. Results of Cox PH regressions modelling incident mortality risk, including interaction terms with time.
