# Supplementary material for: Disentangling the Association of Hydroxychloroquine Treatment with Mortality in Covid-19 Hospitalized Patients through Hierarchical Clustering
Source: J Healthc Eng. 2021 Jun 25;2021:5556207. doi: 10.1155/2021/5556207 (PMC8238578; doi:10.1155/2021/5556207)
Supplement: Supplementary Materials — Figure S1. Cross-patient dissimilarity matrix based on Gower distance. Figure S2. Optimal number of clusters (k) to apply in the hierarchical clustering analysis. Figure S3. Check for basic assumptions of Cox PH models. Table S1. Contingency table of Covid-19 cluster by disease severity. Table S2. Contingency tables of Covid-19 clusters by drugs used for treatment. Table S3. Results of Cox PH regressions modelling incident mortality risk, including interaction terms with time. [file 5556207.f1.docx]

**Supplementary Methods**

**Figure S1. Cross-patient dissimilarity matrix based on Gower distance.**


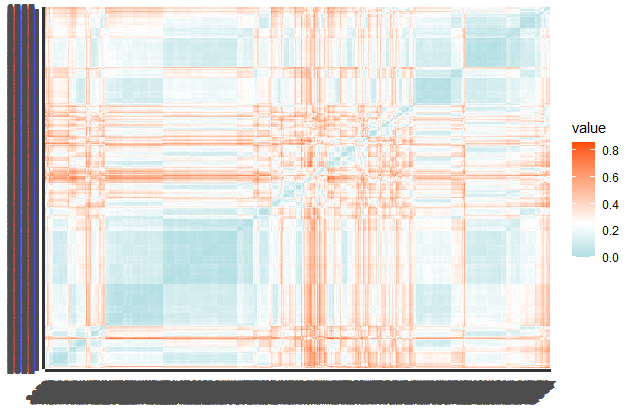


Each squared dot represents a pairwise comparison between patients.

**Figure S2.** Optimal number of clusters (k) to apply in the hierarchical clustering analysis.

The optimal number of clusters (k) was computed through the Silhouette method. The high value of the “Average silhouette width” (> 0.2) indicates that each element is well matched to its cluster and poorly matched to neighboring clusters. The highest peak in the plot is taken as the optimal number of clusters (k=2, in this case).

**Figure S3.** Check for basic assumptions of Cox PH models.

**a)**

**b)**

Test for **a)** lack of influential observations (using dfbeta residuals) and **b)** proportionality of hazards assumptions (Schoenfeld residuals) are reported. No dfbeta residuals >2/sqrt(N) were observed, suggesting the absence of outlier observations. Although no anomaly in the trends of Schoenfeld residuals vs follow-up time was observed, we detected statistical evidence for violations of the PH assumption, which prompted us to perform analyses also including an interaction term with time (see below).

**Supplementary Results**

**Table S1.** Contingency table of Covid-19 cluster by disease severity.

| Covid-19 disease  severity vs clusters | | **Patients’ cluster** | |
| --- | --- | --- | --- |
|  |  | **Cluster 1 – low risk** | **Cluster 2 – high risk** |
| **Disease severity** | Asymptomatic/mild | 524 (13.5%) | 39 (8.2%) |
|  | Non-severe pneumonia | 1577 (40.6%) | 124 (26.0%) |
|  | Severe pneumonia | 996 (25.7%) | 200 (41.9%) |
|  | ARDS | 784 (20.2%) | 114 (23.9%) |
| Total N (with available classification) | | 3,881 | 477 |

Only participants with disease severity classification available were included in the table.

Abbreviations: ARDS = Acute respiratory distress syndrome.

**Table S2.** Contingency tables of Covid-19 clusters by drugs used for treatment.

| **Drug** | **Use** | **Cluster 1**  **low risk** | **Cluster 2**  **high risk** | **Fisher Exact Test (p)** |
| --- | --- | --- | --- | --- |
| **Hydroxychloroquine** | **No** | **861 (22.6%)** | **139 (30.1%)** | **4.6×10^-4^** |
|  | **Yes** | **2,948 (77.4%)** | **322 (69.9%)** |  |
| **Anti-hypertensive drugs** | **No** | **2,464 (63.9%)** | **136 (28.7%)** | **<10^-15^** |
|  | **Yes** | **1,391 (36.1%)** | **337 (71.3%)** |  |
| Anti-interleukin-6 antibody | No | 3,218 (85.0%) | 398 (84.9%) | 0.95 |
|  | Yes | 568 (15.0%) | 71 (15.1%) |  |
| Remdesivir | No | 3,731 (97.4%) | 449 (96.5%) | 0.29 |
|  | Yes | 99 (2.6%) | 16 (3.5%) |  |
| **Lopinavir/Darunavir** | **No** | **2,015 (53.6%)** | **271 (58.8%)** | **0.04** |
|  | **Yes** | **1,746 (46.4%)** | **190 (41.2%)** |  |
| **Corticosteroids** | **No** | **2,286 (65.6%)** | **250 (56.3%)** | **1.5×10^-4^** |
|  | **Yes** | **1,199 (34.4%)** | **194 (43.7%)** |  |

Absolute count of drug users and relative frequency within each cluster are reported. Significant comparisons (p < 0.05) are highlighted in bold. Only participants with medication information available were included in each table.

**Table S3.** Results of Cox PH regressions modelling incident mortality risk, including interaction terms with time.

| **Model** | **Cluster**  **2 vs 1** | **HCQ**  **Yes vs no** | **Cluster*HCQ** |
| --- | --- | --- | --- |
| Death ⁓ Cluster | **3.71**  **[3.15-4.38]** | **-** | **-** |
| Death ⁓ Cluster + HCQ | **3.93**  **[3.31-4.66]** | **0.69**  **[0.59-0.81]** | **-** |
| Death ⁓ Cluster + HCQ + Cluster*HCQ | **2.77**  **[2.33-3.28]** | **0.59**  **[0.50-0.69]** | **1.81**  **[1.49-2.20]** |
| Death ⁓ HCQ (within “low risk” cluster) | **-** | **0.61**  **[0.51-0.72]** | **-** |
| Death ⁓ HCQ (within “high risk” cluster) | **-** | 1.06  [0.77-1.48] | **-** |

**Associations between incident mortality risk, Covid-19 clusters identified and use of Hydroxychloroquine (HCQ), in the three incremental models tested in the total sample, as well as within each cluster. Hazard Ratios** with 95% confidence intervals (HR [CI]) are reported. **Significant HRs (p < 0.05) are highlighted in bold.**
